# Proliferative verrucous leukoplakia/multifocal leukoplakia in patients with and without oral submucous fibrosis

**DOI:** 10.4317/medoral.26106

**Published:** 2023-11-22

**Authors:** Vinay Hazarey, Karishma Madhusudan Desai, Saman Warnakulasuriya

**Affiliations:** 1 Emeritus Professor, Datta Meghe Institute of Medical Sciences, Wardha, India; 2 Postdoctoral Fellow, Tokyo Dental College, Tokyo, Japan; 3 Emeritus Professor, King’s College London and the WHO Collaborating Centre for Oral Cancer, London, UK

## Abstract

**Background:**

Oral submucous fibrosis (OSF) and proliferative verrucous leukoplakia (PVL) are established as oral potentially malignant disorders. Dual pathology of the two conditions is not commonly encountered in clinical practice. This study aims to present a case series of multifocal leukoplakia in patients with and without OSF to outline the clinical behavior and challenges in the management of this high-risk group in clinical practice.

**Material and Methods:**

We retrospectively analyzed cases of six Indian patients (four with OSF) managed over a period of 5.5 to 13 years at the Government Dental College, Nagpur. Patient data consisting of age, gender, medical history, habits, clinical findings, and biopsy reports were recorded at the initial visit. During follow-up visits, the clinicopathological data were reassessed. When surgical intervention failed to arrest the disease or when surgery was contraindicated metronomic therapy with Folitrax 15 mg once a week and Celecoxib 100mg twice daily was initiated.

**Results:**

All patients developed PVL after the initial pathology diagnosis of OSF or oral leukoplakia. Initial lesions were either homogenous or non-homogenous leukoplakia. All patients developed multiple recurrences, regional or systemic metastasis. Despite thorough interventions, the patients died of, or with the disease.

**Conclusions:**

The occurrence of two or more oral potentially malignant disorders poses challenges in patient management and possibly presents a higher risk of malignant transformation. More clinical trials are necessary to assess the benefits of metronomic therapy for patients diagnosed with aggressive PVL concurrently found with OSF.

** Key words:**Proliferative verrucous leukoplakia, multifocal leukoplakia, Oral submucous fibrosis, oral squamous cell carcinoma, mouth cancer.

## Introduction

Oral submucous fibrosis (OSF) is a common oral potentially malignant disorder (OPMD) encountered in most parts of South Asia and Western pacific regions (1). OSF was first described in early 1950s in five Indian women living in South Africa and subsequently there has been a growing literature on this disease following epidemiologic studies conducted in the Indian subcontinent starting from 1965 (2) and later from Taiwan and the Pacific regions (3). The prevalence of OSF in India is reported to be from 0.2-2.3% in males and 1.2-4.6% in females (4). The attribuTable cause of OSF is chewing betel quid with or without tobacco. Areca nut, the primary ingredient in betel quid, is the major risk factor for causation of OSF (5-7). Patients diagnosed with OSF may present with concomitant OPMDs such as oral leukoplakia and erythroleukoplakia (8). These lesions may arise in the background of OSF associated with areca nut use or due to other risk factors such as smokeless tobacco use. Paymaster (9) first made the observation that patients diagnosed with oral squamous cell carcinoma (SCC) at the Tata Memorial Hospital, Bombay, India have had underlying OSF for many years. A 10-year follow up study of a large cohort of Indian subjects confirmed that OSF precedes the development of oral squamous cell carcinoma (10). In 2005, based on such evidence OSF was classified as an OPMD (11).

Proliferative verrucous leukoplakia (PVL) is an OPMD which presents protean clinical manifestations. It is a progressive, multifocal disorder with a high rate of malignant transformation. Though field cancerization is implicated for the progressive course of PVL, its etiopathogenesis is different to the other commonly encountered OPMDs such as conventional leukoplakia (12). Studies describing the dual pathology of multifocal leukoplakia arising in OSF patients is meagre. Concurrent presence of OSF with PVL (multifocal leukoplakia) has not been reported in the literature. The aim of this study is to present a case series of PVL in OSF and leukoplakia patients and to outline the challenges in the management of this high-risk group in clinical practice.

## Material and Methods

Cases for this study were selected from a large cohort of OSF patients managed at the Government Dental College Nagpur from the year 2005 till 2017 and further followed up until 2021. New patients presenting during this period with a clinical diagnosis of OSF or leukoplakia and who later developed multifocal leukoplakia were selected and entered to this study. All patients had a pathological diagnosis certified by an experienced oral pathologist. Diagnostic histopathology criteria used in the study are consistent with the binary grading system and the recently published WHO guidelines (13,14).

Demographic information and lifestyle factors of included cases (betel quid chewing, smoking, smokeless tobacco, and alcohol use) were obtained from clinic charts. A clinical examination of the oral cavity and neck palpation was conducted at presentation by a specialist oral pathologist and during every visit to the clinic. Zain *et al* (15) criteria were used for the diagnosis of OSF and Warnakulasuriya *et al* (11) criteria for diagnosis of oral leukoplakia and erythroplakia. For patients presenting with multi focal oral leukoplakia, Hansen *et al* (16) criteria were used to confirm the diagnosis of PVL.

Biopsies were obtained from representative areas for confirmation of clinical diagnosis and if cancer was suspected.

Management : As the range of clinical presentations varied from leukoplakia or OSF, multiple biopsies were taken to evaluate pathological status. Surgical intervention included excision along with medical management using systemic steroids. Depending on the grade of OSF and patients’ systemic condition oral metronomic chemotherapy (with Folitrax 15 mg once a week and Celecoxib 100mg twice daily) was initiated (17). Imaging investigations were undertaken on follow up visits to investigate spread of disease, nodal involvement and metastasis to bone. Broadly, the standard treatment given to patients who developed malignancies included surgery with neck dissection, radiotherapy and palliative metronomic chemotherapy. The patients were closely followed up using a standard protocol (available on request from the authors), with safety precautions taken during Covid-19 pandemic. Despite periodic telephonic follow-up and photographic evaluations, there were unprecedented complexities in patient management sometimes affecting the prognosis during the Covid-19 pandemic.

Ethical permission for the study was obtained from the Institutional Ethics Committee (IEC-GDCHN/09/52) and all patients provided their consent to be entered to the study.

## Results

Out of six cases, four had the features of dual pathology of OSF and PVL. Table 1 summarizes their clinicopathological features. The case series included three males and three females, with the mean age at diagnosis 59.8 ± 14.1 years. Five of six cases had deleterious tobacco and areca nut/quid chewing habits. Patients were followed-up from minimum 5.5 years to 13 years. In all four OSF cases at least one patch of leukoplakia or several multifocal leukoplakia transformed to SCC or verrucous carcinoma within an average period of about six years. All six cases showed recurrences of excised leukoplakias. Five cases presented with metastasis; three showed regional lymph node metastases, and two showed distant metastasis to either mediastinum, lungs. brain, or vertebrae.

**Table 1:**
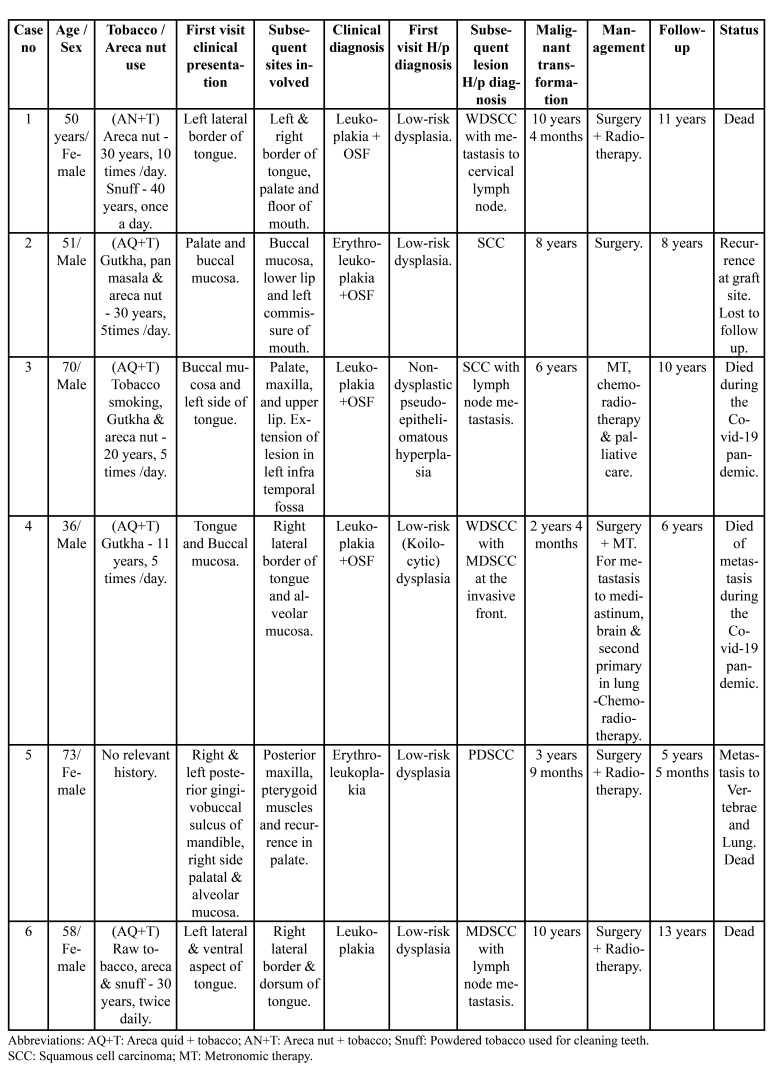
Demographic, clinico-pathological, management and follow-up data.

All six patients died either of oral malignancies or with pre-existing disease. The case series is briefly described below:

- Case 1

**Figure 1:**
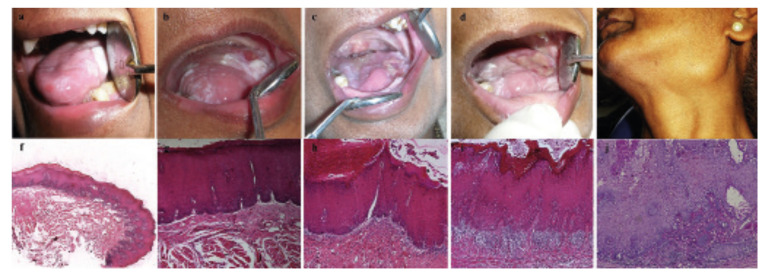
Clinical and histological changes from first visit (2005) to final follow-up (2016) illustrating spread of lesion and nodal involvement in case 1. a) Intraoral image of homogenous leukoplakia of tongue observed on the first visit (2005); b) Post-excision multiple white patches observed during follow-up (2006); c) Subsequent post-excision recurrent lesions (2013); d) Fourth follow-up (2013) showing extensive white patches covering the entire tongue; e) Enlarged lymph node observed on follow-up (2016); f) Hematoxylin and eosin (H & E) stained image (4x) of initial biopsy (2005) showing low-risk dysplastic lesion. g,h,i) Subsequent biopsies (2006 - 2013) showing high-risk dysplasia (H & E; 10x); j) Follow-up biopsy (2016) showing squamous cell carcinoma. (H & E; 10x).

A 50-year-old-female patient first presented with OSF and homogenous leukoplakia over the left lateral border of the tongue. The patient had a history of burning sensation and restricted mouth opening for five years. She had used tobacco snuff for 40 years for cleaning teeth and chewed areca nut for 30 years, ten times/day. Clinical examination revealed marble-like blanched oral mucosa, restricted mouth opening (20mm), deviated uvula, and palpable fibrous bands on the labial and buccal mucosa, and soft palate. Biopsy of buccal mucosa confirmed the diagnosis of OSF. Excisional biopsy of the white patch on the left side of tongue showed a low-risk dysplasia. One year postoperative, a recurrence was noted at the excised site of tongue and subsequently new patches of leukoplakias occurred on the right lateral border of tongue, palate, and the floor of the mouth. Follow up biopsy showed progression from a low- to high-risk dysplastic lesion, to eventual malignant transformation of the leukoplakia patch that recurred following excision. Pathology revealed a well-differentiated SCC (Fig. 1). Fine needle aspiration cytology (FNAC) of a palpable cervical node was positive for regional metastasis. Based on clinicopathological findings, wide surgical excision with radical neck dissection was performed, and treatment with adjuvant radiotherapy (32 cycles) was undertaken. After rigorous follow-up and surgical interventions of two further SCCs over eleven years, the patient died with recurrent neck metastasis.

- Case 2

**Figure 2:**
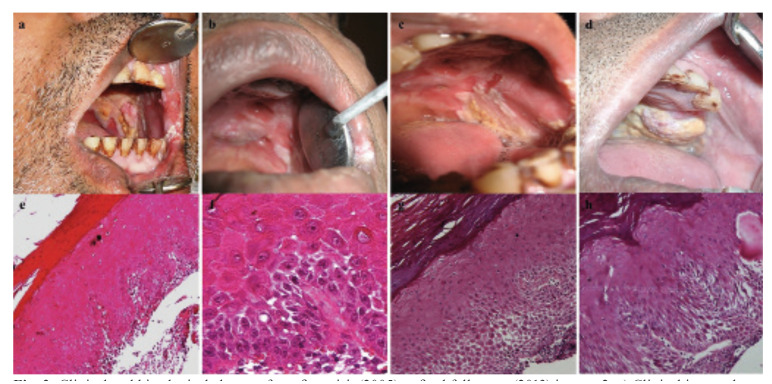
Clinical and histological changes from first visit (2005) to final follow-up (2013) in case 2; a) Clinical image showing restricted mouth opening and intraoral extensive verrucous and red and white patches on the palate and buccal mucosa (2005); b,c) Clinical images showing recurrent lesions over the palate, buccal mucosa and commissure of mouth (2007,2008); d) Follow-up images (2013) showing white patches and a verrucous growth; e,f) Photomicrograph showing low- to high-risk dysplasia in biopsy of initial (H & E; 10x) and recurrent lesions (H & E; 40x); g,h) Photomicrograph of final biopsy showing early change to microinvasive squamous cell carcinoma (H & E; 10x).

A 51-year-old male patient initially presented with clinical features of OSF and a history of reduced mouth opening over five years. The patient gave a history of gutkha and pan masala chewing for 30 years, five times a day. Clinical examination showed a proliferative palatal growth with erythroleukoplakia patches of the palate and buccal mucosa measuring 20 x 30mm. The mouth opening measured 25mm. Biopsy of the non-homogenous leukoplakia patch was indicative of low-risk dysplasia. After two years, three biopsies were taken from lesions involving the palate and buccal mucosa that showed verrucous appearance and had microscopic features of high-risk dysplasia. Recurrence of white patches and verruciform growth involving the lower lip and left commissure of mouth were seen in the fifth and eighth-year follow-up, respectively. Biopsy of verruciform patch showed features of SCC (Fig. 2). Due to the progressive clinical course, wide excision with hemi mandibulectomy and graft-based reconstruction was undertaken. In the same year, another recurrence occurred at the graft site, and the patient was lost to follow-up (presumably died of the disease).

- Case 3

A 70-year-old male patient initially presented with OSF and diffuse erythroleukoplakia patches on the buccal mucosa and left tongue. The patient gave a history of gutkha chewing and restricted mouth opening for six years. On examination, fibrous bands were observed in the oral cavity. Left alveolar mucosa showed papillomatous, exophytic lesions of 10 x 10mm. Microbial investigations of the lesion were positive for *Candida albicans* with no evidence of the human papillomavirus infection.

**Figure 3:**
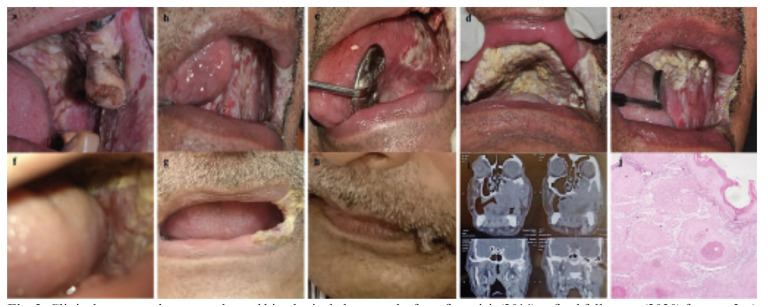
Clinical, computed tomography and histological photographs from first visit (2014) to final follow-up (2020) for case 3. a) Intraoral photograph showing partially edentulous maxilla and diffuse erythroleukoplakia lesions over the alveolar mucosa and buccal mucosa. Posterior palate shows papillomatous exophytic lesions (2014); b,c,d) Post-excision extensive recurrence of erythroleukoplakia over the buccal mucosa, left tongue and palate (2017); e,f) In the sixth year (2020) extensive verrucous and ulcerated lesions were observed in the posterior maxilla and buccal mucosa; g,h) Extraoral photo showing restricted mouth opening (18mm) and involvement of commissure of lip; i) Computed tomography (2020) showing destructive lesions involving the left maxilla, orbit, infra temporal fossa and zygoma; j) Photomicrograph of final biopsy (2020) showing change to squamous cell carcinoma (H & E; 10x).

Excisional biopsies of the multiple non-homogenous leukoplakia lesions showed non-dysplastic, pseudoepitheliomatous hyperplasia and non-specific inflammation. The patient refused surgery and opted for metronomic chemotherapy using Folitrax 15mg once a week and Celecoxib 100mg twice daily. The patient was asymptomatic for two years but presented with extensive proliferative red and white lesions on his tongue in the fourth year. In the sixth year, the patient tested positive for COVID-19, and the metronomic therapy was discontinued. The same year, the patient presented with a proliferative verrucous lesion involving the palate and multiple ulcerated lesions of the left maxilla, buccal mucosa, and upper lip. This was accompanied by paresthesia of the left infraorbital region and a restricted mouth opening of 18mm. Biopsy indicated malignant transformation to well-differentiated SCC. Computed tomography showed a destructive bony lesion involving the left maxilla extending up to the orbit, infratemporal fossa, and zygoma, measuring 5.8 cm x 5.4 cm (Fig. 3). The regional lymph nodes were positive for metastasis. As the patient was reluctant to undergo surgical intervention, he was referred to a medical oncologist for chemoradiotherapy and palliative care, but he died of the disease.

- Case 4

**Figure 4:**
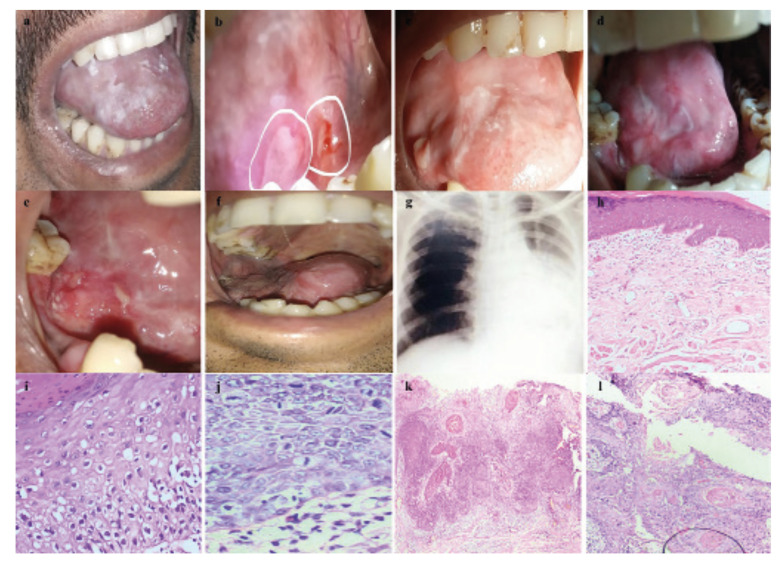
Clinical, radiograph and histological presentation from first visit to final follow-up (2017 to 2022) for case 4. a) Initial clinical presentation showing restricted mouth opening and homogenous white lesion covering the tongue (2017); b) New red-white lesions involving ventral surface of tongue (2017); c) Post-excision follow-up showing new nodular lesion over the anterior part of tongue (2018); d,e) Intraoral photographs showing new red-white lesions over the right lateral border of tongue (2019); f) Post reconstruction follow-up (2020) images showing reduced mouth opening and involvement of alveolar mucosa; g) Chest x-ray showing opacities in mediastinal region and lungs suggestive of distant metastasis (2022); h) Photomicrograph of initial biopsy showing low-risk dysplasia (H & E; 10x); i) Higher magnification of initial biopsy showing koilocytic change in the epithelium (H & E; 40x); j) Photomicrograph of third biopsy showing high-risk dysplasia with increased number of mitotic figures (H & E; 40x); k) Photomicrograph of subsequent follow up (2019) showing squamous cell carcinoma (H & E; 10x); l) Photomicrograph of final biopsy (2020) showing well-differentiated squamous cell carcinoma (H & E; 10x).

A 36-year-old male patient presented with features of OSF and leukoplakia on the tongue. The patient gave a history of gutkha chewing five times/day for 11 years. On clinical examination, the mouth opening was 28 mm, and the mucosa appeared marble-like with palpable fibrous bands on the buccal mucosa. The tongue showed depapillation and flat, non-scrapable, white lesion of 3 cm x 3 cm in size. The biopsy of this homogenous leukoplakia showed low-risk dysplasia. The epithelial cells also exhibited koilocytic change. Metronomic chemotherapy comprising Folitrax 15mg/week and Celecoxib 100mg/day was used to manage the condition. After four months of therapy, the first lesion regressed, but a new lesion on the ventral and right lateral border of the tongue was observed. Excisional biopsy of the lateral border of tongue lesion showed high-risk dysplasia. On follow-up, the two red-yellow lesions measuring 10mm in size recurred at the excision site. Histologically, the lesions showed microinvasive SCC. After 16 months, the patient opted for alternate medicine therapy for the electrocautery-induced allergic reaction and discontinued the use of metronomic therapy. In the second year of follow-up, diffuse red granulated lesions on the right lateral border and alveolar mucosa were seen. MRI showed a few enlarged homogeneously enhancing lymph nodes of levels I-V. Histologically, the lesion showed features of well-differentiated SCC (Fig. 4). Wide surgical excision of the tongue with selective mandibulectomy and neck dissection was undertaken. Adjuvant metronomic therapy was re-initiated. Histological examination of excised tissue showed well to moderately differentiated SCC. Subsequently, the patient developed lung, mediastinal, and brain metastasis along with recurrence in the PMMC flap. The patient underwent chemoradiotherapy for distant metastasis but died of the disease.

- Case 5

A 73-year-old female patient initially presented with clinical features of multiple non-homogenous patches and reported a burning sensation in the oral cavity. The patient gave no history of tobacco or areca nut consumption habits. Clinical examination showed multiple flat red and white and verrucous white patches of approximately 15mm involving the right and left posterior gingivobuccal sulcus of the mandible and right side of palate extending to maxillary alveolar mucosa. White lichenoid-appearing lesions were noted on the alveolar mucosa of the left side of maxilla. The biopsy of the right maxillary palatal region revealed low-risk dysplasia with architectural changes. After three years of follow-up, ulcero-proliferative lesions involving the right palatal region extending to mid palatine raphe of 2 x 2 cm and similar lesions in the left mandibular gingiva buccal sulcus in the 34 to 36 region were noted. Incisional biopsy of the palatal lesion revealed low-risk dysplasia, while gingival buccal sulcus showed high-risk dysplasia. On excision, the right palatal lesion histologically showed features of Verrucous Carcinoma. After six months, the palatal lesion recurred, which showed features of well-differentiated SCC. Wide surgical excision of the left posterior maxilla, including pterygoid muscles and left selective neck dissection (I-IV levels), was undertaken. A biopsy of the second recurrence showed poorly differentiated SCC. Hemi-maxillectomy with radical neck dissection and radiotherapy of 66 Gy/ 33cycles over 6.5 weeks was administered. Postoperatively, the patient showed trismus with pain. Adjuvant metronomic therapy using Folitrax 15mg/week and Celecoxib 100mg/daily was prescribed. After six months, the trismus worsened, and distant metastasis to the vertebra (D11 - osseous) and lungs was noted. Radiotherapy of 8 Gy for five cycles was instituted; however, the patient died after two months of radiotherapy.

- Case 6

A 58-year-old female patient presented with diffuse leukoplakia patches and a burning sensation for seven years. The patient gave a history of chewing raw tobacco ten times a day and cleaning teeth with snuff for over 30 years. On clinical examination, diffuse, flat, non-scrapable, white patches suggestive of homogenous leukoplakia were observed on the tongue at left lateral and ventral aspect, measuring about 30 x 30mm. Biopsy of the tongue lesion revealed low-risk dysplasia. Intralesional steroids were administered, and partial remission was observed after three months. After a year, the lesion recurred on the ventrolateral part of the tongue. A second biopsy was undertaken which showed an epithelium with verrucous appearance and features of low-risk dysplasia. Regression was noted four months after surgical excision. In the second year of follow-up, a non-scrapable white patch on the right lateral border of the tongue was observed. The biopsy showed non-dysplastic epithelium. Post-excision, a small, elevated nodule was noted on the left dorsum of the tongue. The excisional biopsy of the lesion was histologically diagnosed as SCC. After three years, the patient showed an extensive lesion traversing from the left to the right border involving the ventral to dorsum aspect of the tongue. Incisional biopsy showed well-differentiated SCC, and the patient underwent hemiglossectomy with radical neck dissection. The excisional specimen showed moderately differentiated SCC with lymph node metastasis. After two years of surgery and radiotherapy of 66 Gy, 33 cycles for 6.5 weeks, the patient died of the disease. The progressive PVL lesions showed transformation from a low-risk dysplastic lesion to SCC over a span of thirteen years.

## Discussion

An association of OSF with other OPMDs such as leukoplakia or erythroplakia has been reported sporadically (8) but to our knowledge concurrent presence of OSF with PVL has not been reported in the literature. We report here a case series from Nagpur, India that describes development of multifocal leukoplakia in the background of pathologically confirmed OSF or conventional leukoplakia.

PVL was first described by Hansen *et al* based-on clinico-pathological findings in a cohort of 30 patients attending a hospital clinic in San Francisco (16). New diagnostic criteria were developed by Lafuente *et al*. (12) and would be useful for future follow up studies. Most reports on PVL found in the literature are either from USA or Europe (12,18,19) and the clinical aspects of the disease are not well characterized among patients from Indian origin. For this reason, our clinical descriptions in this current series may have some educational merit to the readers.

The novel aspect of this report is the development of PVL in the background of OSF in four patients described here. These two conditions have different developmental aspects in terms of etiopathogenesis. OSF is primarily caused by exposure to areca nut (in betel quid) most likely induced by arecoline which is an alkaloid found in areca nut (6,20). The etiology of PVL mostly reported in European or North American populations remains unknown and is not considered to be tobacco-induced. It is therefore of scientific interest to question why this group of OSF patients developed PVL over a long period of follow up. OSF is also considered primarily as a collagen disorder as its pathology reveals deposition of collagen in the submucosa (21,22). The deposition of collagen in OSF is caused by the activation of fibroblasts by cytokines e.g., TGF β that is released because of chronic inflammation induced by the action of arecoline (23). On the other hand, PVL is primarily a mucosal disorder with underlying proliferation of the oral epithelium leading to multiple areas of keratoses often with verrucous hyperplasia. The co-existence of these two pathological entities affecting the oral cavity therefore remains an enigma.

Both PVL and OSF are considered to have a high frequency of malignant transformation; 40-50% of patients with PVL eventually develop oral malignancies (12,24,25) while around 10 % of patients with OSF may develop SCC (26,27). This perhaps explains the aggressive nature of the condition with dual pathology observed in patients described here as majority developed oral malignancies with extensive metastatic disease with fatality.

Patients with OSF mostly come from medically underserved communities. Early diagnosis and follow up of these cases are often a challenge due to limited health literacy of affected individuals and their poor access to health care. Limited mouth opening associated with the disease makes it difficult for the clinician to systematically examine the oral cavity leading to misdiagnosis. Fast growing proliferative lesions could easily be missed if the follow up system is not efficient. In the present series, all cases underwent multiple biopsies and needed major surgical interventions including neck dissection at a specialized center. This case series provides evidence of frequent recurrence of oral leukoplakia in OSF patients pointing to the need to develop a fail-safe system in oral medicine clinics to follow up patients with OSF.

There are no guidelines for management of either of these two clinical conditions or for those with this dual pathology. Reviews on the management of OSF found no effective medical therapies (8,28). Due to large surface areas affected by the disease with multifocal leukoplakia surgical intervention is not possible without damaging vital structures and losing function. In advanced cases we therefore applied Metronomic chemotherapy in an off-label trial comprising Folitrax 15mg/week and Celecoxib 100mg/day to manage the condition in 3/6 patients. In metronomic therapy, a lower dose, typically varying from one-tenth to one-third of the maximum tolerated dose, is administered frequently to maintain a low concentration of the drug in the plasma. Instead of directly killing the proliferating cells, the drugs in metronomic therapy suppress their growth mainly by inhibiting angiogenesis in the micro-environment of the affected zones and modulating the immune response against the proliferating cells (29). Low-cost oral metronomic chemotherapy has recently been tried as a palliative intervention in patients with recurrent, metastatic, inoperable head and neck carcinoma in an open label randomized clinical trial (30). In our series the two drugs were given in oral form, which is more convenient for OSF patients and had a lower cost compared with intravenous form used in conventional chemotherapy. This regime suited our patients who were mostly from underprivileged backgrounds. There is no effective treatment to manage either of these two conditions (28). This is the first-time metronomic therapy has been applied to treat advanced OSF complicated by proliferative leukoplakia. Our series is too small to statistically evaluate its effectiveness but provides some insight to its potential use and for a future well planned clinical trial for OSF patients. Until novel medical therapies for management of OSF are developed risk reversal by counselling on cessation of betel-quid habit among affected individuals is advocated.

## Conclusions

We describe a novel case series of six Indian patients who had multifocal leukoplakia, out of which four patients had preexisting OSF. Considering the rising incidence of OSF in India, cases with concomitant occurrence of multifocal leukoplakia need meticulous screening and long-term follow-up. The elusive biological behavior and progressive malignant transformation of this dual pathology poses a serious challenge to the management team. We highlight the challenges in management of this aggressive dual pathology. After surgical inventions had failed during advanced stages, metronomic chemotherapy was introduced as a palliative measure.
